# The assessment of dietary diversity score and associated factors among pregnant women of Batu district, Southern Ethiopia, 2021: a community-based cross-sectional study

**DOI:** 10.1097/MS9.0000000000000239

**Published:** 2023-02-06

**Authors:** Genanew K. Getahun, Sindew M. Ahmed, Abinet B. Degif, Mekonnen G. Haile

**Affiliations:** a Menelik II Medical and Health Science College, Kotebe Metropolitan University; b Addis Ababa Medical and Business College, Addis Ababa, Ethiopia

**Keywords:** dietary diversity, pregnant women, Southern Ethiopia, undernutrition

## Abstract

**Background:**

Malnutrition remains a global problem, particularly in sub-Saharan Africa, where Ethiopia is located. During pregnancy, inadequate nutritional diversification increases the risk of unfavorable maternal and fetal outcomes. Therefore, the aim of this study was to assess the dietary diversity score and associated factors among pregnant women in Batu district, Southern Ethiopia, in 2021.

**Methods:**

A community-based cross-sectional study was conducted among randomly selected 594 pregnant women. Data were collected with a two-stage sampling technique through face-to-face interviews. The data were coded and entered into Statistical Package for the Social Sciences (SPSS) version 23. Bivariate and multivariable logistic regression analyses were applied to identify independent predictors of dietary diversity.

**Results:**

The magnitude of the unmet minimum dietary diversity score among pregnant women was 356 (59.9%). Furthermore, pregnant women with no formal education [adjusted odds ratio (AOR)=3.46; 95% CI: 1.99, 5.66], poor by the wealth index (AOR=2.23, 95% CI: 1.33, 3.73), having five or more children (AOR=1.75, 95% CI: 1.14, 2.71), multigravida (AOR=2.18, 95% CI: 1.34, 3.56), and pregnant women from only male-headed households (AOR=4.46, 95% CI: 2.86, 6.94) were associated with an unmet minimum dietary diversity score among pregnant women.

**Conclusion:**

The prevalence of unmet minimum dietary diversity scores among pregnant women was found to be high. Moreover, low dietary diversity was linked to pregnant women with no formal education, multigravida, having more than five family members, male-headed households, and being poor by household wealth. As a result, nutritional diversity education should be prioritized, and health experts should provide guidance on dietary diversity and family planning services.

HIGHLIGHTS

- Malnutrition remains a global problem, particularly in sub-Saharan Africa.
- During pregnancy, inadequate nutritional diversification increases the risk of unfavorable maternal and fetal outcomes.
- The prevalence of unmet minimum dietary diversity score of pregnant women was found to be high (59.9%).

## Introduction

Pregnancy is a critical period that places an additional burden on women’s nutritional requirements to satisfy the metabolic and physiological demands of both the mother and the growing fetus^1^. Healthy nutrition is associated with a lower chance of developing chronic diseases, healthier pregnancy and delivery, newborn development, and child health^2^. Dietary diversity during pregnancy is critically important; hence, it has been proven to affect pregnancy and birth outcomes^3^–^5^. According to the Food and Agricultural Organization, dietary diversity is a qualitative indicator of food intake that shows households’ access to a variety of foods and the adequacy of a person’s diet in terms of nutrients^6^.

Nearly two billion people are suffering from micronutrient deficiencies globally^3^. For pregnant women, who are typically nutritionally susceptible due to the physiological demands of pregnancy, undernutrition is particularly common^5^,^7^. In sub-Saharan Africa, diets are primarily made up of starchy carbohydrates, with little or no animal products and few fresh fruits and vegetables^8^. Particularly in Ethiopia, pregnant women are considered nutritionally vulnerable due to a variety of socioeconomic factors, such as low dietary intakes, unequal food distribution, improper food preparation, dietary taboos, infectious diseases, and a lack of nutritional knowledge^9^–^13^. During pregnancy, a lack of nutritional diversity increases the risk of unfavorable maternal and neonatal outcomes^14^–^17^. Evidence in Ethiopia suggests that the appropriate nutritional practices for pregnant women range from 19.9 to 40.1%^18^,^19^.

The Ethiopian government is working to end all kinds of malnutrition by 2030, including meeting the globally agreed targets for addressing the nutritional needs of teenage girls, pregnant women, lactating women, and older people by 2025^20^,^21^. Despite the availability of some literature elsewhere in Ethiopia^11^,^22^, the evidence is insufficient, particularly in the rural part of Southern Ethiopia, a region with drastically diverse cultures and economic realities. Therefore, the goal of this study was to determine the dietary diversity score and associated factors of pregnant women in Batu district, Southern Ethiopia.

## Methods

### Study area and population

The study was conducted in the Batu district of Southern Ethiopia. It is located 162 km away from Addis Ababa, Ethiopia. Based on the estimated projections from the 2007 national census, the district had an estimated population of 192 359^23^. Among them, 49% were males and 51% were females, including 6656 (3.46%) pregnant women. The district was divided into 26 kebeles with 39 257 residential houses and an average household size of five people. There were six health centers, five private clinics, and 23 health posts. The study was conducted from 1 August to 30 September 2021. The work has been reported in line with the Strengthening The Reporting Of Cohort Studies in Surgery (STROCSS) criteria^24^.

A community-based cross-sectional study was conducted among randomly selected pregnant women. Pregnant women who were 18 years of age or older and had been registered with a family folder by health extension workers were included. Those who had been registered in the family folder and had residency relocated outside of the selected district were excluded.

### Sample size and sampling procedure

The sample size was determined using a single population proportion formula based on the following assumptions: a proportion (*p*) of 38.8%^25^, a 95% confidence interval (CI), and a 5% margin of error (*d*). The calculated sample size was 365. Finally, including a design effect of 1.5 (two-stage sampling) and a possible nonresponse rate of 10%, it gives 605.

**Figure 1:**
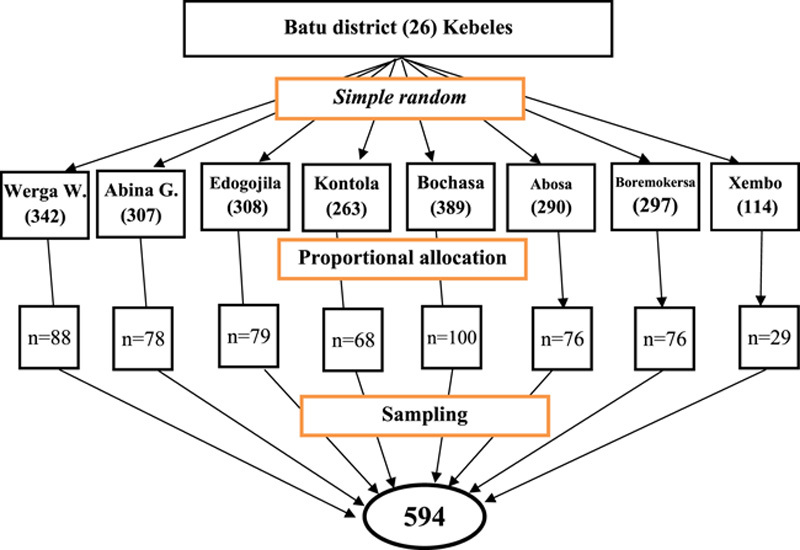
Schematic presentation of the sampling procedure.

The study was carried out with a two-stage sampling technique. Batu district has a total population of 192 359 people and 6656 pregnant women among its 26 kebeles (the smallest administrative unit in Ethiopia). Eight kebeles were selected randomly by using the lottery method. The sample size was allocated proportionally for each selected kebele based on the expected number of mothers among the total eligible population. In the second stage, the households were identified using a pregnant women’s registration list available at health posts. Finally, the starting households were determined by a simple random sampling technique (Fig. 1). Whenever two women were available within a household, one was selected using the lottery method.

### Study variables and definitions

Minimum dietary diversity score for women (MDD-W) was regarded as the outcome variable, and sociodemographic factors (age, place of residence, marital status, mother’s education, income, etc.), maternal characteristics (the frequency of antenatal care (ANC) visits, the birth interval, the number of meals, etc.), anthropometric measurement (BMI), mid-upper arm circumference (MUAC), and environmental factors (drinking water source, latrine accessibility) were also taken as predictor variables. Dietary diversity was coded as 1 for individuals who met the minimum dietary diversity requirement and 0 for those who did not.

*Undernutrition* refers to the nutritional status of pregnant women, which is influenced by nutrient intake and utilization and determined by a MUAC less than 210 mm^21^.

*Dietary diversity*: the number of food items ingested by pregnant women. It was calculated out of 14 food groups, which include grains, white roots, tubers, and plantains; pulses; nuts and seeds; dairy; meat, poultry, and fish; eggs; dark green leafy vegetables; and other vitamin-rich fruits and vegetables that are nutrient-dense.

*The pregnant women’s MDD-W* was based on the consumption of five or more food groups of the 14 food items as reported by the pregnant women through 24-hour dietary recalls^26^.

*A ‘male-headed household’* is a term used to describe a person who provides the household’s principal source of income and food^27^.

### The data collection and analysis

Data were gathered through face-to-face interviews with a standardized questionnaire adapted from previous Food and Agricultural Organization studies^6^. Before data collection, the questionnaire was translated into ‘Afaan Oromo’ and then returned to English to ensure consistency and maintain conceptual equivalence. The Food Insecurity Experience Scale (FIES) was used to assess individual food security^6^. Maternal nutritional health was determined using the MUAC. A nonstretchable MUAC tape was used to measure the MUAC. It was measured three times on the same day, using calibrated equipment and established methodologies.

For data gathering, four data collectors were assigned. A 1-day training was scheduled and provided for both the data collectors and supervisors. Before obtaining anthropometric measurements, the instruments were calibrated. The quality of the data was ensured by pretesting the questionnaire and strict data collection supervision. The language consistency of the questionnaire was reviewed, and a pretest was conducted before the actual data collection period to estimate the time required for each data collection.

The response was coded and entered into the computer using EPI Info version 7.1 and then exported to Statistical Package for the Social Sciences (SPSS) version 25 for further analysis. A frequency table, chart, and figures were used to summarize and present the descriptive data. Bivariate logistic regression analysis was used to identify potential candidate variables for the multivariable logistic regression analysis. Those variables having a *P*-value less than 0.25 during the bivariate analysis were entered into the multivariable logistic regression analysis. The result of the final multivariable logistic regression model was expressed in terms of adjusted odds ratios (AORs) and 95% CIs. Statistical significance was declared with a *P*-value less than 0.05.

### Patient and public involvement

Throughout the proposal development, data collection period, and analysis, pregnant women and members of the public provided free support and advice for the researchers regarding ethical issues and tips on how to communicate their findings to a broad audience in a way that the general public can understand and benefit from.

## Results

### Demographic and socioeconomic characteristics

This study covered a total of 594 pregnant women, with a response rate of 98.2%. The average (SD) age of the study participants was 27.6 (4.42) years, with the bulk of 287 (48.3%) falling between the ages of 25 and 34 years. The vast majority of respondents (398, or 67.0%) came from rural areas; 558 (93.9%) were married and lived together; and 31 (5.2%) had polygamous husbands. When it came to the educational level of the pregnant women, 181 (30.5%) had no formal education, while 351 had (59.1%) primary schooling. The majority of the study participants were housewives, with 526 (88.6%) and 513 (86.4%) having a farmer spouse. Male-headed or male-dominated families accounted for the majority of the participants (455, or 76.6%). In terms of family size, the mean (SD) was 4.9 (±1.94), with 410 families (69%) having five or fewer children and 184 families (31%) having five or more children. In all, 284 (47.8%), 208 (35%), and 102 (17.2%) of the households were poor, middle-class, and wealthy, respectively (Table 1).

**Table 1:** Socioeconomic and sociodemographic characteristics.

Variables	Category	Frequency, *N* (%)
Age	18–24 years	210 (35.4)
	25–34 years	287 (48.3)
	35 or above years	97 (16.3)
Residence	Urban	196 (33.0)
	Rural	398 (67.0)
Marital status	Unmarried	16 (2.7)
	Married	558 (93.9)
	Divorced	11 (1.9)
	Separated	9 (1.5)
Husband had polygamy	No	563 (94.8)
	Yes	31 (5.2)
Education status	No formal education	181 (30.5)
	Primary school (1–8)	351 (59.1)
	Secondary school (9–12)	45 (7.6)
	College and above	17 (2.9)
Occupational status of lactating mothers	Housewife	526 (88.6)
	Private worker	20 (3.4)
	Government employee	19 (3.2)
	Merchant	28 (4.7)
	Student	1 (0.2)
Occupational status of husband	Farmer	513 (86.4)
	Private worker	10 (1.7)
	Government employee	18 (3.0)
	Merchant	50 (8.4)
	Student	3 (0.5)
Household’s wealth	Poor	284 (47.8)
	Medium	208 (35.0)
	Wealthy	102 (17.2)
Number of people living in family	≤5	410 (69.0)
	>5	184 (31.0)
Head of household	Male	455 (76.6)
	Female	139 (23.4)

### Obstetric and nutritional characteristics

The majority of the pregnant women (399, or 67.2%), 546 (91.9%), and 513 (86.4%) had ANC follow-up, health institution delivery, and postnatal care follow-up, respectively. Multigravida and multipara were found in nearly half of the respondents, 318 (53.5%) and 308 (51.9%), respectively. Only 20 (3.4%) of the study participants had twin children (Table 2). Moreover, using MUAC measurements, 112 (18.9%) of the participants had recently lost weight.

**Table 2:** Obstetric characteristics of study participants.

Variables	Category	Frequency, *N* (%)
Breastfeeding twins	No	574 (96.6)
	Yes	20 (3.4)
ANC follow-up	No	195 (32.8)
	Yes	399 (67.2)
Gravida	One	134 (22.6)
	Two	142 (23.9)
	Multi	318 (53.5)
Para	One	141 (23.7)
	Two	145 (24.4)
	Multi	308 (51.9)
Abortion in the last 6 months	No	459 (77.3)
	Yes	135 (22.7)
Place of child born	Health institution	546 (91.9)
	Home	48 (8.1)
Mode of delivery	Vaginal delivery	574 (96.6)
	Cesarean delivery	20 (3.4)
PNC follow-up	No	81 (13.6)
	Yes	513 (86.4)
Abortion in the last 6 months	No	459 (77.3)
	Yes	135 (22.7)
History of diarrhea in the past 2 weeks	No	582 (98.0)
	Yes	12 (2.0)
Cough and difficulty of breathing	No	586 (98.7)
	Yes	8 (1.3)

ANC, antenatal care; PNC, postnatal care.

### Dietary diversity of pregnant women

**Figure 2:**
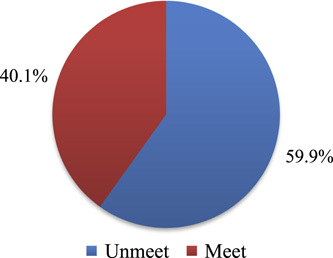
The level of dietary diversity score among pregnant women in Batu district, Southern Ethiopia, 2021.

The mean minimum dietary diversity score of pregnant women (MDDS-W) (SD) was 4.4 (±1.39). Breakfast was reported by 592 (99.8%) of the study participants, and lunch was reported by 589 (99.3%). Foods from grains, white roots, tubers, and plantains were ingested by the majority, 591 (99.5%). Dark green leafy vegetables were consumed by 463 pregnant women (78.1%) (Table 3). The magnitude of the unmet minimum dietary diversity score among pregnant women was 356 (59.9%) [95% CI: 56.0, 63.8], whereas 238 (40.1%) were met by MDDS-W in the Batu district (Fig. 2).

**Table 3:** Dietary diversity scores of respondents.

Food groups	Frequency, *N* (%)
Breakfast	592 (99.8)
Morning snack	170 (28.7)
Lunch	589 (99.3)
Evening snack	551 (92.9)
Grains, white roots, tubers, and plantains	591 (99.5)
Pulses (beans, peas, and lentils)	360 (60.6)
Nuts and seeds	70 (11.8)
Milk, cheese, yogurt, or other milk products	159 (26.9)
Meat, poultry, and fish	43 (7.3)
Eggs from chicken	32 (5.4)
Dark green leafy vegetables	463 (78.1)
Vitamin A-rich vegetables, roots, and tubers	100 (16.9)
Tomato, onion, green-pepper, fresh corn.	95 (16.1)
Apple, avocado, banana, blackberry, grapes, lemon	100 (16.9)

### Factors associated with an unmet minimum dietary diversity score

Using bivariate logistic regression analysis, variables with a *P*-value less than 0.25 were selected first, and age, polygamy, education status, household wealth, number of people living in the family, gravida, ANC follow-up, and head of household were statistically associated with an unmet minimum dietary diversity score and were candidates for multivariable logistic regression analysis.

Finally, the multivariable logistic regression analysis revealed that pregnant women with no formal education, multigravida, having more than five children, pregnant women from male-headed households, and being poor by household wealth were all predictors of unmet MDDS-W.

As a result, pregnant women with no formal education were more than three times as likely to be unmet for the minimum dietary diversity score (AOR=3.36, 95% CI: 1.99, 5.66) than those with a high school diploma or higher. When compared to their counterparts, pregnant women with more than five children (AOR=1.75, 95% CI: 1.14, 2.70) or multigravida (AOR=2.18, 95% CI: 1.34, 3.56) were nearly twice as likely to have unmet MDDS-W. Pregnant women from poor households were about two times (AOR=2.23, 95% CI: 1.33, 3.74) more likely to be exposed to having their MDDS-W needs unmet than pregnant women from wealthy households. Furthermore, pregnant women from solely male-headed households had a fourfold (AOR=4.46, 95% CI: 2.86, 6.94) increased risk of not meeting the minimal dietary variety score (Table 4).

**Table 4:** Factors associated with unmet dietary diversity.

	MDDS-W			
	Unmet	Met		
	*N* (%)	*N* (%)	COR (95% CI)	AOR (95% CI)
Age				
18–24 years	110 (52.4)	100 (47.6)	1.119 (0.690, 1.815)	1.091 (0.641, 1.856)
25–34 years	182 (63.4)	105 (36.6)	1.763 (1.070, 2.906)	1.694 (0.982, 2.920)
35 or above years	64 (66.0)	33 (34.0)	1	1
Husband had polygamy				
Yes	25 (80.6)	6 (19.4)	2.920 (1.179, 7.231)	2.555 (0.960, 6.801)
No	331 (58.8)	232 (41.2)	1	1
Education status of respondents				
No formal education	116 (70.3)	49 (29.7)	2.663 (1.680, 4.222)	3.365 (1.999, 5.666)*
Elementary school	168 (60.9)	108 (39.1)	1.522 (1.008, 2.298)	1.352 (0.859, 2.127)
High school or above	72 (47.1)	81 (52.9)	1	1
Household’s wealth				
Poor	187 (65.8)	97 (34.2)	2.347 (1.481, 3.720)	2.233 (1.335, 3.737)*
Medium	123 (59.1)	85 (40.9)	1.332 (0.921, 1.928)	1.132 (0.753, 1.699)
Wealthy	46 (45.1)	56 (54.9)	1	1
Number of people living in family				
≤5	230 (56.1)	180 (43.9)	1	1
>5	126 (68.5)	58 (31.5)	1.700 (1.178, 2.454)	1.753 (1.137, 2.703)*
Gravida				
One	78 (54.9)	42 (31.3)	1	1
Two	92 (68.7)	64 (45.1)	1.797 (1.098, 2.941)	2.180 (1.336, 3.558)*
Multi	186 (58.5)	132 (41.5)	1.555 (1.014, 2.384)	1.818 (1.056, 3.130)*
ANC follow-up				
Yes	227 (56.9)	172 (43.1)	1	1
No	129 (66.2)	66 (33.8)	1.481 (1.037, 2.116)	1.260 (0.848, 1.872)
Head of household				
Female	45 (34.6)	85 (65.4)	1	1
Male	311 (67.0)	153 (33.0)	3.107 (2.098, 4.601)	4.459 (2.865, 6.941)*

* Statistically significant on multivariate analysis, *P*-value less than 0.05.

ANC, antenatal care; AOR, adjusted odds ratio; COR, crude odds ratio; MDDS-W, mean minimum dietary diversity score of pregnant women.

## Discussion

The current study revealed that the level of unmet minimum dietary diversity score was 59.9% [95% CI: 56.0, 63.8]. This finding was consistent with research findings from Angecha districts in Ethiopia 57%^13^, Axum town in Tigray, Ethiopia 62.7%^28^, and Ghana 56%^3^. It was, however, greater than reports from Western Ethiopia 37.1%^29^ and Zambia 38%^30^. In contrast, it was lower than prior study reports from Ethiopia, Vietnam, and Bangladesh, at 38.8%, 32.4%, and 28.1%, respectively^25^,^31^,^32^. This disparity could be due to socioeconomic differences as well as seasonal, cultural, and traditional variations in food preparation among the research populations.

According to the results of multivariable logistic regression analysis, pregnant women with no formal education were three times less likely to meet the minimal dietary variety score than women with a high school diploma or higher. This was in line with study findings from Ethiopia’s Tigray region^25^ and Patiala City, northwestern India^33^. This might be due to the fact that pregnant women with a higher level of education might have more job chances, which can bring a better income. Besides, they could have a good command of resources autonomously and adhere to prescribed procedures.

In addition, when compared to their counterparts, pregnant women with a family size of more than five and pregnant women with multigravida were exposed to an increased risk for unmet MDDS-W. This was supported by research findings from Southern Ethiopia^13^, western Hill Nepal^34^, and northeast Ethiopia^11^. This could be due to the fact that pregnant women are most vulnerable to MDDS, as they are more likely to have low MDDS-W due to frequent round deliveries and low family encouragement^25^,^35^.

Pregnant women from low-income families and pregnant women from homes with just a male head of household were more likely to have their MDDS-W needs unmet than their counterparts. This was in line with research findings from Ghana^3^, Northern Ethiopia^25^, and Western Hill Nepal^34^. This could be related to participants’ occupational positions, which could impair dietary diversity owing to a lack of information about how they prepare their everyday dishes, as they are familiar with conventional food preparation methods. Because of the cultural dominance of the male or husband in the household, they had no right to access their resources and money to acquire what they needed.

As a limitation, the feeding practice may be biased by recall and social desirability. In addition, since the study design is cross-sectional, it does not establish a causal or temporal relationship between the outcome variable and independent factors. We made no attempt to account for seasonal variations in the food supply, which can affect MDDS-W.

## Conclusion

The current study revealed that there is a high level of unmet MDDS-W. Moreover, unmet dietary diversity scores were linked to pregnant women with no formal education, multigravida, having more than five family members, male-headed households, and being poor by household wealth. Therefore, to reduce the risk of malnutrition during pregnancy and adverse birth outcomes, nutritional diversity education should be prioritized, and health experts should provide guidance on dietary diversity and family planning services.

## Ethical approval

Ethical approval was first secured from the research and ethical review board of Addis Ababa Medical and Business College, with approval number 19/2021. Then a permission letter was obtained from Batu district health officials. Written informed consent was obtained from each study participant immediately before the interview. The ethical principles outlined in the Declaration of Helsinki guide the entire research process, which states that ‘it is the physician’s or researcher’s responsibility to promote and protect the health, well-being, and rights of patients, including those who participate in medical research.’

## Patient consent

Written informed consent was obtained from each study participant immediately before the interview. Moreover, all the study participants were informed that their participation was voluntary and of the potential benefits, confidentiality, and possibility of withdrawing from the interview at any time. Confidentiality was assured by blinding the name of the patient profile or any specific characteristics, instead by using a code and medical registration number.

## Sources of funding

The study has no funding source.

## Author contribution

G.K.G.: conceptualization, software, data curation, visualization, investigation, writing – original draft, review, and editing; S.M.A.: supervision and writing – review and editing; A.B.D.: conceptualization, methodology, software, and writing – review and editing; M.G.H.: conceptualization, software, visualization, and writing – original draft.

## Conflicts of interest disclosure

The authors declare they have no competing conflicts of interest.

## Research registration unique identifying number (UIN)

1. Name of the registry: Research Registry.
2. Unique identifying number or registration ID: researchregistry8290.
3. Hyperlink to your specific registration (must be publicly accessible and will be checked): Browse the Registry – Research Registry UIN.docx

## Guarantor

All authors will take responsibility for the work, access to data, and decision to publish.

## Data availability

The datasets will be shared upon reasonable request.

## Provenance and peer review

Not commissioned, externally peer-reviewed.
